# Suicide or accident? A psychological autopsy study of suicide in youths under the age of 16 compared to deaths labeled as accidents

**DOI:** 10.1186/1753-2000-6-30

**Published:** 2012-09-12

**Authors:** Anne Freuchen, Ellen Kjelsberg, Berit Grøholt

**Affiliations:** 1 Department of Psychiatry, Faculty of Medicine, University of Oslo, N-0316, Oslo, Norway; 2 Department of Child and Adolescent Mental Health, Sørlandet Hospital, N-4604, Kristiansand, Norway; 3 Centre for Forensic Psychiatry, Oslo University Hospital, N-0407, Oslo, Norway; 4 Institute of clinical medicine, Faculty of medicine, University of Oslo, N-0361, Oslo, Norway

**Keywords:** Children, Adolescent, Suicide, Accident, Suicide intent, Labeling

## Abstract

**Objective:**

In the present paper, we describe suicide in youths under 16 years of age and compare their risk factors for suicide to those of older adolescents as described in the literature. Furthermore, we evaluate the possible mislabeling of suicides as accidents, and vice versa.

**Method:**

We used the data from a nationwide psychological autopsy of youths 15 years and younger who had committed suicide or died in accidents in Norway from 1993 to 2004 (n = 84). We additionally constructed a suicide index to distinguish between the two causes of death.

**Results:**

The young suicide victims presented, with little gender difference, fewer obvious risk factors and less suicide intent than commonly described for older adolescents. The suicide index distinguished quite well between suicides and accidents, with few cases indicating a possible mislabeling, although some suicide cases could have been labeled as uncertain.

**Conclusion:**

In line with previous research, suicides in 11-15-year-olds have many similarities to suicides in older adolescents in terms of external circumstances, but they present less apparent warning signs. In our total sample of 84 deaths, there were few indications of incorrect labeling.

## Background

Adolescents do commit suicide, even if it is rare under the age of 15. The suicide rate in Norway among 10–14 year olds in 2001–2005 was 1.2/100 000 (boys 1.5, girls 0.9/100 000) and decreased in 2006–2009 to 0.6/100 000 (boys 0.7, girls 0.5/100 000). In 2009, the rate was 1.3/100 000, compared to 0.7/100 000 in the USA in the age group 5–14 years according to the Centers for Disease Control and Prevention (http://www.cdc.gov/), decreasing in Norway to 0.3/100 000 in 2010 (http://www.ssb.no). Numerous studies of suicide in older adolescents and young adults exist, but there has been relatively little such research on young adolescents and children [1-4]. Among older adolescents, the most important risk factors for suicide are the presence of a psychiatric disorder, substance abuse, conflict with friend, disruption of a romantic relationship, and a previous suicide attempt [3,5]. In younger adolescents and children, the risk factors are less clear, with lower incidences of diagnosable psychiatric disorder and less substance abuse; furthermore family factors are important. However, the problems or conflicts between child and parents are often perceived to be of minor importance from an adult perspective [3,6,7]. There are differences in typical suicide methods worldwide, as strangulation (Europe), pesticide (Asia) and firearm (USA) dominate country-specific suicide patterns [8], age not taken into account. In his review article, Greydanus [9] noted a general trend in the USA toward strangulation as opposed to using firearms in the 10-19-year-olds. Strangulation is now reported to be the most common suicide method in the youngest age group [3,6,10]. In some cases, strangulation may look confusingly like asphyxia games, which are, also outside the realm of autoerotic asphyxia, contributing to the difficulties of labeling youth suicide [11]. Some studies suggest that the extent of childhood suicide is underestimated, with suicides often misclassified as accidents [12]. Other studies, however, have hypothesized that the opposite may also be a problem [13]. Suicidal intent could help to distinguish between true suicides and accidents, but is less frequently described because suicide notes and verbal communications are less common among younger age groups [1,13].

The purpose of our study was to conduct a nationwide psychological autopsy from 2007 to 2009, on children and adolescents 15 years and younger who committed suicide in Norway between 1993 and 2004, as well as on a group of children of the same age who died in accidents during the same time span. We gathered detailed information on the children and on the circumstances surrounding their deaths [6].

In this paper, we aim to 1) provide more knowledge and detail about suicide among youths 15 years and younger; 2) investigate risk factors for committing suicide in our sample compared with those for committing suicide in older adolescents as described in the literature; and 3) explore the possible mislabeling of events as suicides or accidents in relation to suicide risk factors.

## Material and method

A more complete description of the data and data collection procedure used in our study is provided in a previous article [6]. The labeling of each death as a suicide or accident was made according to the vital statistics obtained from Statistics Norway (SSB). SSB when in doubt, make an effort to determine whether a death was a suicide or an accident, and a labeling as “Undetermined Death” is hardly ever used (http://www.ssb.no/).

### Suicide victims

From SSB, we received the names and addresses of the parents of all 91 residents 15 years and younger whose deaths were classified as suicides in Norway between 1993 and 2004. Among these parents, 42 (46%) agreed to participate in our study. The mean age of the children was 14.3 years (SD 1.1, range 11.7-15.9) of which 16 (38%) were 15-year-olds.

We had no gender information on the 49 non-participants, whose mean age was 13.8 years and not significantly different from that of the participants. The non-participants’ methods of suicide were 76% strangulation, 14% firearm, 4% intoxication, 2% drowning and 4% not specified, which was also not significantly different from the participating group.

### Accident victims

Our comparison group was 10-15-year-olds who died in accidents between 1993 and 2004 in Norway. Natural disasters and events in which the child was a passenger (car, bus, train or boat) were excluded. SSB provided the parents’ names and addresses, and we enrolled 42 (44%) participants. The mean age of the children was 13.2 years (SD 1.6, range 10.0-15.5).

We obtained no gender information on the 54 non-participants in the comparison group. Their mean age was 13.2 years and not significantly different from that of the participants. The accidents among the non-participants were 41% traffic accidents, 13% drowning, 7% fall, 5% strangulation, 4% intoxication, 2% firearm and 28% other or not specified and were not significantly different from those participating.

The group of under-15-year-olds provides some challenges with regard to which term is the most appropriate one; childhood (4–12 years), middle childhood/preadolescent (10–13 years) and adolescents (14–15 years) (Random House Dictionary). As we in our analyses treat them as one group, we have chosen to use the term youths, whereas when we refer to the conversation with the parents, we use the term child, as they did, regardless of the deceased’s age.

### Procedure

The procedure was identical for both groups. First, the parents received an invitation letter, to which they had to respond actively. A reminder letter was mailed 3–4 weeks later. The parents decided whether both or just one of them would participate. They were interviewed in 2007–2009 by the first author, a specialist in psychiatry and child and adolescent psychiatry. The interview included the administration of The Schedule for Affective Disorders and Schizophrenia for School Aged Children (6 – 18 years): Present and Lifetime Version (Kiddie-SADS-PL) and was recorded on audio tape in 68% of the participants. The assessment of psychiatric diagnoses, in accordance with the Diagnostic and Statistical Manual of Mental Disorders 4^th^ edition (DSM-IV, American Psychiatric Association, 1994), was made by the first author using all available information. By blinded assessment, another psychiatrist made the diagnoses independently for 14 randomly selected Kiddie-SADS-PL interviews using the Statistical Package of Social Sciences version 16.0 (SPSS) for the selection. An acceptable reliability was obtained, with a kappa of 0.82.

We obtained the police-reports for 38 (91%) suicides and 28 (67%) accidents, (OR = 4.8, CI = 1.4-16.0, p = .02).

### Measurements

In this article, we assumed the causes of death as given by the SSB to be correct. These are based on information in the death certificate from the hospital/physician and, if performed, the autopsy-report.

We collected the demographic information on the youth and their parents that was valid at the time of the child’s death, including family structure (parents living together or not, siblings or not). We obtained from the parents their description of the death scene, the method of death, where and when it took place (time of day, day and month), who found the deceased and whether the deceased was alive when found. We also asked the parent if a farewell note of any kind was found as well as their opinion as to whether the death was a suicide or an accident. We noted losses that the deceased had experienced throughout life that presumably would have affected her/him emotionally, such as the loss of a family member, friend or other significant relation, a pet or other animal. In addition, we asked if there had been any interpersonal conflict (with parents, school, peers, police or others), and if so, when this was taking place in relation to the time of death. We also noted if the child had been bullied, without grading it further. To get an idea of the youth’s suicidal ideations, we surveyed whether the parents knew that he/she had been interested in the topic of suicide, such as having mentioned it to the parents in different ways and wanting to discuss it. Questions about suicidal behavior (suicidal ideations, threats or attempts) [14] are also included in Kiddie-SADS-PL [15], a semi-structured diagnostic interview with a parent version that was used in an approved Norwegian version after translation and back-translation. Furthermore, the Kiddie-SADS-PL has questions about self-harm and drug use. The parents were asked to grade the mental health of their child on a 4-point scale (very good, good, some problems or serious problems), and the Kiddie-SADS-PL was used to evaluate mental disorders. Depressive symptoms that failed to meet full criteria for depression in the DSM-IV were noted as sub-threshold depression, in accordance with the definition given by Fergusson [16]. In addition, we asked the parents to describe the child’s usual manner and personality traits. They used adjectives that we could easily put into four categories (vulnerable-touchy, worried-anxious, self and socially confident, impulsive-temperamental). Each category was independently rated as being present or not; i.e., the categories were not mutually exclusive. We also recorded the number of suicides in the family’s history and we noted suicides in the local community during the last 2–3 years that the youth had been well aware of.

We further asked about school attendance on the day of death and noted if the child had shown altered behavior shortly before the suicide. The pre-suicide behavior was categorized as positive (calm, happy, helpful, cuddly, etc.), negative (tense, unhappy, sad, irritated, angry, etc.) or normal (as usual).

We obtained information both from parents and hospital records, on any previous or current contact with Child and Adolescent Mental Health Services (CAMHS), any hospitalization, ongoing medication, and type of medicine (antidepressants, antipsychotics, central stimulants, anti-diabetics, or other, i.e., mostly anti-allergic).

The police reports provided additional information including the physical circumstances of the death scene, death date and assumed time of death, which was grouped into four periods: morning, afternoon, evening and night. The months of the year were dichotomized into the bright season (March-August) and dark season (September-February). A medical autopsy had been conducted in 23 (61%) cases of suicide and 14 (50%) accident cases (OR = 1.5, CI = .5-4.1, p = .46). In some reports, there was information about the deceased given by relatives and/or friends. No discrepancies between the police reports and the parents’ information were observed.

The Suicide Intent Scale (SIS) was also used. The SIS consists of two parts [17]. Part I (items 1–8) covers the objective circumstances surrounding the suicide or suicide attempt. It describes three main components: precautions, planning and communications [18], including items used in preparation for the suicide, the manner of its execution, the setting, and any clues given beforehand that could have facilitated or hampered interventions. Part II of the SIS is based on reports from suicide attempters and is therefore not relevant to this study. On the basis of all available information, mainly parents’ account and the police report, each item of Part I was rated 0, 1 or 2, yielding a total range of 0–16. Higher scores on the SIS are indicative of a greater intent to commit suicide.

Based on the hypothesis that some of the suicide victims might not have intended to die and that some accidents actually represented suicides, we constructed a suicide index to see if we could separate the two groups regarding their load of risk factors to suicide. The index is not constructed to be used in other contexts as the psychometric qualities are not tested. It consists of commonly described risk factors and other factors having a possible impact on suicidal behavior. All items were graded 0–1 (no – yes), except Depression and Suicide Attempt, which both were graded 0–2 (no – yes). A score of 2 on Depression excluded a positive score on Sub-threshold Depression. The variable “Any other psychiatric diagnosis” included the diagnoses of Anxiety disorder, Asperger syndrome, ADHD and Conduct disorder. As a result, the index ranged from 0–17, with 17 representing the maximum load of risk factors. The method of death, suicide note, parental uncertainty about the cause of death, and SIS scores were not included in the index, but they were used in the discussion of possible mislabeling.

### Ethics

All required permissions were obtained from the Norwegian Directorate for Health and Social Affairs, the Norwegian Social Science Data Services, the Director General of Public Prosecution, the Directory of Residents, and Statistics Norway. The Regional Committees for Medical and Health Research Ethics approved the study.

### Statistical analyses

All analyses were performed using the Statistical Package of Social Sciences version 16.0 (SPSS Inc., Chicago, IL USA). Pearson chi-square or Fisher’s exact test were used for cross tabulations of categorical variables, and a *t*-test was used to conduct independent group comparisons for continuous variables. The significance level was set at p ≤ 0.05, and not-significant is abbreviated n.s. The suicide victims were analyzed and compared with the accident victims on selected variables.

## Results

### Demographic characteristics

In the suicide group, 28 (67%) parents were married and lived together, 6 (14%) were divorced, 6 (14%) were remarried, and 2 (5%) were living alone/widowed, and these numbers were not significantly different from the accident group: 25 (60%), 14 (33%), 2 (5%) and 1 (2%), respectively. Thirty-six (86%) suicide victims had siblings compared to 37 (88%) in the accident group. Eight parents (19%) of the suicide victims were uncertain as to whether their child’s death was a suicide or an accident, six of which were strangulations and two used firearms. This number was significantly higher (p < .01) than the one parent (2%) of an accident victim who expressed such uncertainty.

### Localization, diurnal and seasonal distribution

Some factors related to the death scene are shown in Table 1. In 18 cases (43%), the suicide was committed at home, five (29%) of which were found in the staircase, and five in their bedroom. Three (7%) accidents happened at home, including one strangulation and one intoxication, both were in the victim’s room, while the third drowned in the bathtub. The suicides occurred more often in the afternoon and during the dark season.

**Table 1:** Gender, age and outer circumstances of the deaths by suicide, accident and gender

	**Suicide**		**Suicide group**			**Accident**		**Accident group**			**Suicide vs. accident**		
	**Boys**	**Girls**	**boys vs. girls**			**Boys**	**girls**	**boys vs. girls**					
	***n%***	***n%***	***OR***	***(CI)***	***p***	***n (%)***	***n (%)***	***OR***	***(CI)***	***p***	***OR***	***(CI)***	***p***
Gender	30 (71)	12 (29)				22 (52)	20 (48)				.4	(.2-1.1)	.12
Age, mean	14.3	14.4			.25	13.7	13.2			.63			<**.01**
SD	1.5	1.2				1.6	1.7						
Strangulation	19 (63)	9 (75)			.72	1 (5)	-						
Hung Freely	11 (58)	5 (56)			.46	-	-						
Both feet on the ground	5 (26)	1 (11)				-	-						
Sitting down	3 (16)	3 (33)											
Firearm	9 (30)	1 (8.3)			.23								
Jumping/falling	1 (3.5)	1 (8.3)			.49	-	1 (5)			.48			
Drowning	1 (3.5)	1 (8.3)			.49	4 (18)	4 (20)			1.00			
Intoxication	-	-				2 (9)	-						
Pedestrian, bicycle	-	-				11 (50)	12 (60)			.55			
Train, tram, subway	-	-				2 (9)	1 (5)			1.00			
Other accident*	-	-				2 (9)	2 (10)			1.00			
Place of death													<**.01**
Family residence	10 (33)	8 (67)	.3	(.1-1.0)	.08	2 (9)	1 (5)**	1.9	(.2-22.7)	1.00			
Family garage	6 (20)	1 (8)	2.8	(.3-25.7)	.65	-	-						
Elsewhere	14 (47)	3 (25)	2.6	(.6-11.7)	30	19 (86)	19 (95)	.3	(.0-3.5)	61			
Time of death	N=41				.94	N=34				.81			**.02**
Morning 6a.m.-14p.m.	3 (35)	2 (17)				7 (11)	4 (29)						
Afternoon 14-18p.m.	16 (57)	6 (50)				3 (15)	4 (29)						
Evening 18-24p.m.	6 (40)	3 (25)				8 (21)	5 (36)						
Night 0a.m.-6a.m.	3 (11)	1 (8)				2 (10)	1 (7)						
Died on a	*n = 40*				.41	*n = 35*				.84			.18
Monday	5 (18)	6 (55)				3 (14)	3 (21)						
Tuesday	5 (18)	1 (8)				0	1 (7)						
Wednesday	5 (18)	1 (8)				4 (19)	3 (21)						
Thursday	8 (29)	1 (8)				3 (14)	2 (14)						
Friday	1 (4)	1 (8)				4 (19)	2 (14)						
Saturday	2 (7)	1 (8)				4 (19)	1 (7)						
Sunday	2 (7)	1 (8)				3 (14)	2 (14)						
Season													
Bright (March-Aug.)	11 (37)	3 (25)	1.0			15 (68)	17 (85)	1.0			1.0		
Dark (Sept.-Feb.)	19 (63)	9 (75)	1.7	(.4-7.8)	.72	7 (32)	3 (15)	.4	(.1-1.7)	.28	6.4	(2.5-16.7)	<**.01**
The deceased found by													
Family member	17 (57)	7 (58)	1.0			5 (23)	1 (5)	1.0			1.0		
Other	13 (43)	5 (42)	.9	(.2-3.6)	1.00	17 (77)	19 (95)	5.6	(.6-52.7)	.19	.1	(.0-.4)	<**.01**
Alive when found													
Yes	5 (17)	1 (8)	1.0			13 (59)	3 (81)	1.0			1.0		
No	25 (83)	11 (92)	2.0	(.2-21.1)	.66	9 (41)	3 (19)	.3	(.1-1.5)	.18	13.0	(4.3-39.1)	<**.01**

*Other accident = fire, explosion during play etc.

** = Residence of a relative.

### Risk factors for suicide

#### Experience of loss and conflict

Half of the losses, see Table 2, were of family members. They had also been in a conflict situation more often than the accident victims. Conflict with parents represented 14 (35%) cases, and the parents perceived most of the conflicts to be of minor severity. Conflicts involved the police (one, 2%), school (six, 14%), friends (six, 14%) or other (four, 10%), and categories were not mutually exclusive. The conflict-suicide interval was short. In 16 of the 24 cases, the suicide happened within hours of the actual reported conflict, whereas three occurred within one week. Five of the youths had long lasting conflicts. The results revealed no gender differences in the experience of loss or conflict. Likewise, there was no difference between suicides and accidents regarding bullying.

**Table 2:** Suicide index. Contributing factors to suicide experienced by the suicide and accident victims

**Contributing factors**	**Suicide victims**	**Accident victims**	**Statistics**		
	***n = positive score (%)***	***n = positive score (%)***	***OR***	***CI***	***p***
Loss	21 (50)	7 (17)	5.0	1.8-13.8	<**.01**
Conflict	24 (59)	5 (12)	10.4	3.4-32.1	**.01**
Bullied	12 (29)	6 (14)	2.4	.8-7.2	.18
Suicide interest	19 (46)	1 (2)	35.4	4.4-282.4	**.01**
Suicide threat	4 (10)	0 (0)			.06
Suicide attempt*	5 (12)	0 (0)			**.03**
Self harm	1 (2)	1 (2)	1.0	.1-16.5	1.00
Mental health problems	14 (33)	5 (12)	3.7	1.2-11.5	**.04**
Sub-threshold depression	8 (16)	1 (2)	9.9	1.2-83.5	**.02**
Depression*	4 (10)	0 (0)			.06
Any other psychiatric diagnosis	6 (14)	3 (7)	2.3	.5-9.9	.31
Vulnerable/touchy	21 (50)	4 (10)	9.5	2.9-31.4	<**.01**
Worried/anxious	7 (17)	1 (2)	7.8	.9-66.6	.06
Self or socially confident**	38 (91)	27 (64)	5.3	1.6-17.7	<**.01**
Impulsive/temperamental	6 (14)	0 (0)			**.03**
Suicide in family or local community	18 (43)	1 (2)	30.8	3.9-245.1	<**.01**

All variables are graded 0 = not present or 1 = present.

*indicates variables graded 0 = not present or 2 = present.

** indicates variable graded 0 = present or 1 = not present.

#### Suicidal behavior

Interest in the topic of suicide, suicide threats, suicide attempts and self-harm is presented in Table 2. The parents described their child’s behavior as somewhat altered shortly before the suicide in 21 (53%) children, which was significantly more often than the 2 (5%) in the accident group (OR = 21.6, CI = 4.6-101.6, p < .01). The altered behavior of the suicide victims was labeled negative in 13 (62%) instances and positive in 8 (38%), without gender differences. Furthermore, two suicide victims had experienced suicide in their family and 16 had experienced suicide in the local community, which was significantly more than the accident victims.

#### Mental health

The parents of the suicide victims perceived their child to have mental health problems significantly more often than the parents of the accident victims (Table 2). In contrast, there were no significant group differences in the fulfillment of Kiddie-SADS criteria for any psychiatric diagnosis. Two youths in each group carried an ADHD-diagnosis, with one suicide victim dead by strangulation and one by a firearm, while both accident victims were traffic accidents. The third accident victim had an Anxiety disorder and the other four suicide victims with a diagnosis in addition to depression, included Anxiety disorder, Asperger syndrome, and conduct disorder, alone or in combinations. The personality traits presented in Table 2, indicate that the suicide victims were significantly more vulnerable and more impulsive than the accident victims, and had a tendency to be more worried, though this tendency was not statistically significant. Examining gender across groups, we found that girls who committed suicide were significantly more vulnerable (OR = 3.5, CI = 1.9-6.3, p = .01) and that they were non-significantly more impulsive (p = .05) than female accident victims. The male suicide victims were significantly less confident (OR = .2, CI = .0-.7, p = .01) and more vulnerable (OR = 5.9, CI = 1.6-21.7, p = .01) than the male accident victims. There were no gender differences within the suicide and accident groups.

### Supplementary information

The number of youths who did not attend school on the day of death was 19 (46%) in the suicide group and 27 (64%) in the accident group (p = .12), but these absences were often explained by weekends or holidays*.* However, nine (21%) suicide victims and one (2%) accident victim were truant on the day they died. There were no significant differences between the groups in mental health treatment received by CAMHS (suicide group 6 (14%), accident group 3 (7%), n.s.) or in somatic department hospitalizations (suicide group 10 (24%), accident group 13 (31%), n.s.). Likewise, there were no differences in ongoing medication and type of medicine (suicide group: 5 (12%), 1 antidepressant, 4 other; accident group: 3 (7%), 1 central stimulant, 2 “other”, n.s.).

The medical autopsies revealed a positive toxicology (alcohol and/or drugs) in one male suicide victim and three male accident victims, with no significant difference between the two groups. Parents had additionally reported that one other male suicide victim had been drinking alcohol, but he was not autopsied. Including him in the analyses did not alter the significance.

### SIS and suicide notes in suicide victims

The mean score on SIS-items 1–8 was 8, range 3–14 (see Table 3). There were no significant gender differences on single items, groups of items or in the SIS total score.

**Table 3:** Comparison of the Suicide Intent Scale, items 1 – 8, between the genders in the suicide group

**SIS item**	**Boys**		**Girls**		**Statistics**
	***n = 29***		***n = 12***		
	**n**	**(%)**	**n**	**(%)**	***p***
1. Isolation					.17
Somebody present	1	(3)	1	(8)	
Somebody nearby	9	(31)	7	(58)	
No one nearby	19	(66)	4	(33)	
2. Timing					.14
Intervention probable	4	(14)	5	(42)	
Intervention not likely	16	(55)	5	(42)	
Intervention unlikely	9	(31)	2	(17)	
3. Precautions					.55
No precautions	10	(34)	6	(50)	
Passive precautions	15	(52)	4	(33)	
Active precautions	4	(14)	2	(17)	
4. Acting to get help					.64
Notified helper	2	(7)	0		
Contacted	2	(7)	1	(8)	
No contact	25	(86)	11	(92)	
5. Final acts					.18
None	21	(72)	10	(83)	
Some arrangements	2	(7)	2	(17)	
Completed arrangements	6	(21)	0		
6. Active preparation					.85
None	8	(28)	4	(33)	
Minimal/moderate	14	(48)	6	(50)	
Extensive	7	(24)	2	(18)	
7. Suicide note					1.00
Absence of note	16	(55)	7	(58)	
Presence of note	13	(45)	5	(42)	
8. Communication					.62
None	14	(48)	4	(33)	
Ambiguous	10	(35)	6	(50)	
Marked	5	(17)	2	(17)	
SIS Total score, mean	8.4	SD=3.1	7.0	SD=3.2	.22
Precautions, mean 1 + 2 + 3	3.6	SD=1.4	2.7	SD=1.4	.06
Planning, mean 5 + 6 + 7	2.3	SD=2.0	1.7	SD=1.9	.32
Communication, mean 4 + 8	2.5	SD=.7	2.8	SD=.8	.28

A suicide note, defined as any written message, short message service or letter, was left by 18 (43%), (5 (42%) girls, mean age 13.9; range 11–15, and 13 (43%) boys, mean age 14.0; range 12–15). This frequency was statistically equivalent between the genders. The length of the notes varied from two words on a post-it sheet to two-page letters and school essays.

### The suicide index

In our sample, one death by strangulation was labeled as an accident by SSB. The police report in this case was missing. Another strangulation, labeled suicide by the SSB, was labeled as an accident by the police. In this paper, this death is treated as a suicide.

**Figure 1:**
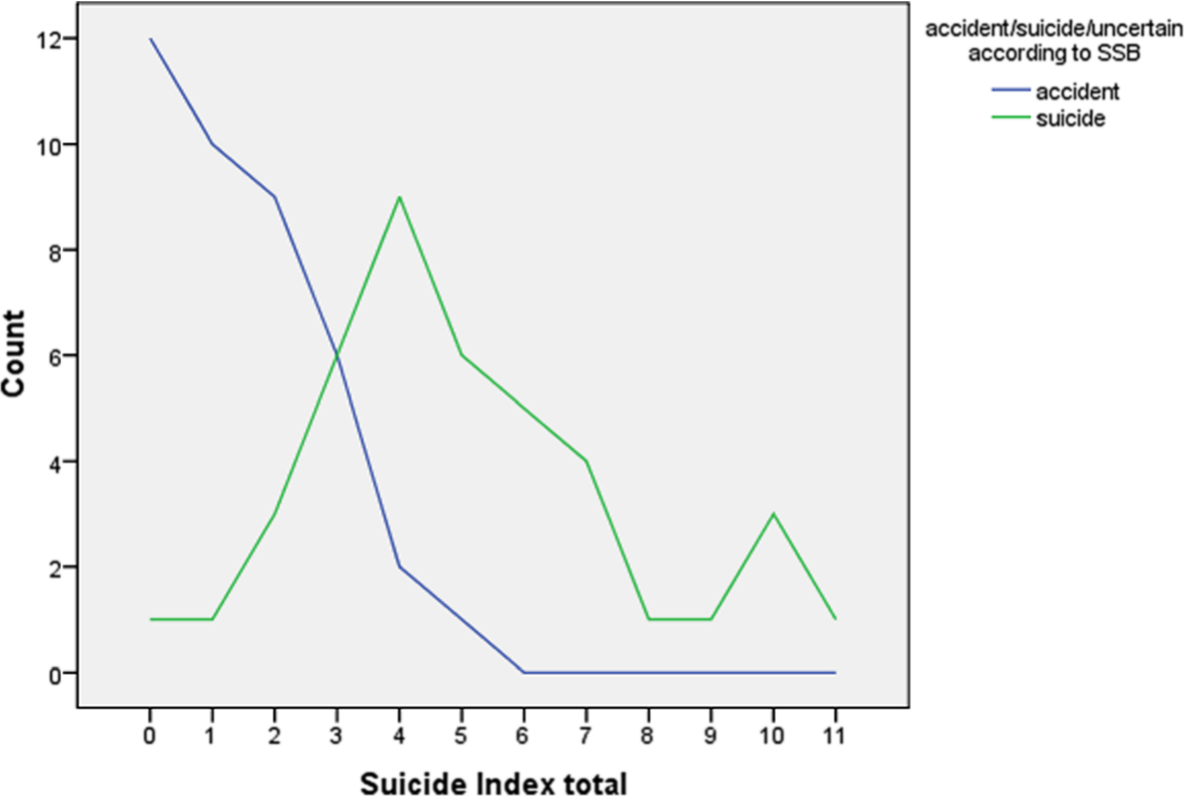
The load of risk factors acording to Suicide Index.

All factors that constitute our suicide index are given in Table 2. Figure 1 displays the distribution of the total index-score in the two groups. There is an intersection between the lines, showing the number of suicides with an index of three or lower (n = 11), and the number of accidents with an index of three or higher (n = 9), with number three as the area of overlap.

Using all available information, we examined each one of these 20 cases in relation to factors that could indicate a risk for mislabeling in the official statistics of suicide vs. accident.

The 11 suicides with a suicide index below four (SIS mean 7.1, range 3–13) were as follows:One jumped from an elevated height, and the jump seemed well planned in every way (SIS = 10).Two victims used firearms with shots to the head (SIS 7 and 13). Of these two, one (SIS = 7) was used to handling a rifle, had plans for the future and was a popular guy; however, he was both impulsive and fearless. There were no signs of planning and no observed precipitating conflicts. He had been talking to a friend on the phone, telling him he would go to school, but he subsequently did not turn up. It is difficult to determine with certainty whether this event was a suicide or an accident, and thus, it could be labeled uncertain. The other firearm victim (SIS = 13) left a note. The parents labeled both deaths as suicide.

Eight suicides incorporated strangulation. Four of them hung fully suspended with both feet above the ground (SIS mean 7, range 5–9), and three of these left suicide notes. In the fourth case, we had too little information to evaluate the labeling, and the police report was not found. Four strangulation victims sat down (SIS mean 5, range 3–8). In two of these cases, the parents expressed uncertainty as to the labeling (SIS 3 and 5, respectively, and both with suicide index = 2). Both of these girls were in trouble with their parents at the time, not wanting to do as they were told and maybe intending more to scare than to die. They lived in the same local community, and the ways in which’s their suicides were committed was identical. The events took place three years apart; thus, the second girl should in principle have known about the lethality of the method.

There were six more cases in addition to the two mentioned above, where the parents expressed uncertainty as to the labeling of suicide. Four of these cases were strangulations, of which one hung fully suspended (suicide index = 5, SIS = 8), while three had both feet on the ground (suicide index mean 4.3, range 4–5, SIS mean 6, range 3–10). In the other two cases, the victim had used firearms. In one of these, the parent gave insufficient information, and the police report was missing, rendering it impossible to score the suicide index and the SIS. The second one had a suicide index = 4 and SIS = 7. The authors’ best estimate was that both were suicides.

The same examination was performed on the nine accidents having a suicide index of three or higher (suicide index mean score 3.1, range 3–4, all had SIS = 0). Of these, five involved pedestrian or bicycle accidents, two were tram/train accidents, one was a fall from a cliff, and one was related to intoxication (cannabis in combination with Ecstasy). The following factors contributed to their suicide index scores: loss 5, conflict 4, suicide interest 1, one or more personality traits 11, bullied 4, and the same 3 scored on both mental health problems and any other psychiatric diagnosis. However, when evaluating the circumstances and all known information, all nine deaths were, according to the authors’ best estimate, correctly classified as accidents.

## Discussion

The suicides of under-16-year-olds in our study had many similarities with the suicides of older adolescents, as described in the research literature. External circumstances hardly differed, but the younger ones presented fewer risk factors and less known intent. The suicide index, constructed for this study to assess potential mislabeling of suicides as accidents, and vice versa, distinguished effectively between the two groups. The extent of mislabeling was small in our sample.

### The suicides

The overrepresentation of boys (71%) in the suicide group is in accordance with studies in other Western countries [3,19,20] but in contrast to Turkey [21], where 71% of suicide victims 15 years and younger were female. Likewise, Chinese suicide victims have a female-to-male ratio close to 1.0 [22]. The explanation as to why boys as well as men commit suicide more often than females is complex and poorly understood. The possible link to high testosterone levels, associated with a higher degree of aggression towards others or self, has been investigated by Perez-Rodriguez [23]. He found no support for the putative role of high testosterone levels as a biological marker of suicidal behavior among adult males. Like Perez-Rodriguez, we found no overrepresentation of aggressive or temperamental personality traits among the male suicide victims. Aggression, a risk factor to suicide, is associated with low CSF 5-HIAA in children and adolescents [24]. However, we had no information on level of testosterone or CSF-5-HIAA.

Like Beautrais and Shaw [10,20], we found that a greater proportion of suicides (60%) took place in the family residence or in the garage used daily by the parents. This has also been found previously in adolescents under the age of 19 [25].

The suicides in our study occurred most frequently during the work week, in the afternoon, after school and before the parents were expected home from work, a time of day children often will be on their own. This is in contrast to the findings of Beautrais [10], who found that suicides tended to take place while family members were present in the house or on the property. The time of day of committing suicide in our sample may reveal intentions to die because these children were likely alone and would not be disturbed. The time and location chosen would secure quick discovery by someone dear. On the other hand, the same circumstances could also be an expression of ambivalence, because it includes the possibility of being rescued.

There are few studies on the seasonality of youth suicides, and the findings are inconclusive. In our sample the suicides occurred more often in the dark season, in line with McCleary who found an unimodal seasonality peak in fall/winter for young males [26], whereas Goren found no seasonal differences [21], and Dervic found the greater number of suicides during April, May, October, and November [19].

The altered behavior observed prior to suicide in 67% of the suicide victims may indicate that the decision to commit suicide had been reached and presented some type of relief. In suicidology, this phenomenon is known among adult victims; however, it is rarely mentioned in the literature on children and adolescents [4].

### Risk factors

#### Mental health

We found 20% of our suicide victims had diagnosable mental disorders, which is relatively low. Psychological autopsy studies in the United States of adolescents under 16 and 17 years of age [1,7] showed a higher prevalence of psychiatric disorders, 60% and 83%, respectively, although lower prevalence than studies that included 15-20-year-olds [7,27]. These studies of 15-20-year-olds showed more than a 90% prevalence rate of psychiatric disorders. The lower prevalence of psychiatric disorders in children compared to older adolescents is an important factor in explaining the lower rates of suicide in children under 16 years of age [1].

Depressive disorder, specifically, was found in only 10% of our sample, whereas Brent [1] found a mood disorder in 43% of his sample in an autopsy study of under-16-year-olds, and Grøholt [3] identified 29% in under-15-year-olds in her investigation of official records. We should note that there are studies that suggest Norwegian parents may tend to underreport emotional disorders in their children [28], which may be an explanation to the low numbers in this autopsy study. The time span between the child’s death and the interview period may also have increased the parents’ tendency not to report depression. International studies show that depression has a higher prevalence in older adolescents [20,27,29]. In his recent study, Gibbons [30] found that when clinical depression is present, the severity of the depression in youths is strongly related to suicide risk. However, while antidepressant medication can reduce depression severity, he found that it had no effect on suicide risk. Thus, an important task is to identify different types of psychopathology, aggressive impulsive traits and other factors contributing to suicide among children.

#### Conflicts

One third of our suicide victims had been bullied. Klomek found an association between frequent bullying at age eight and later suicide attempts and completed suicides up to age 25 and that the later suicidal behavior varied by sex [31]. Kaltiala-Heino found that suicidal ideation was more common among 14–16 –year-olds who were involved in bullying [32]. Still, the association between bullying and completed suicide is not yet fully understood.

A conflict with parents, school, peers or authorities is thought to be a stressor contributing to suicide, and this was indeed found in 60% of our sample. This is in line with the autopsy-findings of Brent [1], which identified 51% of suicide victims who had experienced parent–child conflict. In her review of coroners’ files of children less than 15 years old, Beautrais [10] estimated that 71% had been in trouble that was considered to be a precipitant to suicide. However, conflicts and stressors described in autopsy studies are often categorized differently, and the age groups included vary. Hence, the results are difficult to compare [1,33,34]. Conflict with parents is most often described in young victims, whereas boy/girlfriend conflicts and disruption of romantic relationships are more often cited in older adolescent suicide victims [1].

We found a brief conflict-suicide interval in our data, most often within hours. This is in accordance with Shaffer’s findings [35], although he did not define “brief”. Marttunen [34] found that in older adolescents, all conflicts considered precipitants had occurred within a week preceding the suicide. Greydanus [36] suggested that the mental immaturity of children may contribute to their fate. That is, when faced with a major life crisis, from their point of view, suicide may become an immediate option as a solution to their overwhelming problems. Our study supports this assumption.

#### Loss

Broken relationships and interpersonal losses leaving a marked emotional footprint were experienced by 50% of the suicide victims in our study. Likewise, Gould [33] found that 54% had an experience of loss in her autopsy of suicides in a sample of victims younger than 20 years of age. She defined loss as the death of a relative or friend, disruption of a relationship or a recent separation, much like our definition but without specifying whether pets were involved. Loss and conflict were experienced significantly less often by the accident victims than suicide victims, which gives us reason to believe that both factors might have contributed to suicide. Variability in labeling and in definitions of loss makes comparisons difficult, both across autopsy and non-autopsy studies [3,10,34].

#### History of suicide in the victim’s environment

Over 40% of our sample had experienced suicide previously, either in the family or in the local community. This finding is partially supported by Beautrais [10], who examined coronial files of 61 suicides of youths younger than 15 years of age and found that 15% had experienced a recent death in family, while 10% had a history of suicide in a parent, sibling or cousin. In our study, we included suicide in the local community, which may explain why our numbers are higher. Qin and Runeson, among others, found that suicide in family members are associated with suicide, and Brent and Wender found a familial link between suicide and impulsive aggression [37-40]. However, in our sample of suicide victims, only two had experienced suicide in the family, whereas 16 had experienced suicide in the local community, among peers or neighbors. Mercy [41] found no evidence of prior exposure to others’ suicidal behavior being a risk factor for nearly lethal suicide attempts among children and young adults. Knowledge about the suicide of a respected family member, a peer or neighbor, may induce an acceptance of suicide, and combined with a possible inadequate understanding of the meaning of suicide and death [9,42-44] may give the youth fewer objections to such an action.

### Possible mislabeling

One of the goals of this paper was to look for the possible mislabeling of suicides and accidents. In addition to the factors included in the suicide index, we examined suicide notes (diaries, essays) indicating some type of suicidal planning, school attendance on the day of the suicide, altered behavior prior to the suicide and probable grade of impulsiveness.

None of the accidents gave any indications of being actual suicides that had been mislabeled. In the one accidental strangulation, the parents did express some uncertainty, but after evaluating the case and using all available information, the labeling as accident seemed correct.

None of the 31 suicides having a high suicide index (above 3) showed any indication that they had been accidents that were mislabeled, although the parents were doubtful in five of these cases. In one of those five instances, the information was too incomplete for a sufficiently comprehensive evaluation. Of the 11 suicides with a low risk-load on the suicide index, there was one shooting incident that was ambiguous as to whether it was an accident or a true suicide. Most indications suggested that the four strangulations in which the victims were found fully suspended were suicides. Three of those appeared well planned, while the fourth was most likely an impulsive act. One of the strangulation victims who was sitting down had left an undated note that bequeathed his assets. The two who were in trouble with their parents at the time showed high degrees of impulsiveness, making their parents assume that their child had intended to scare rather than to die; consequently, a labeling of uncertain could have been considered. The fourth showed all the signs of being an accident, reflecting the conclusion of the police report: the boy strangulated himself while he was waiting for his friend. Thus, in three strangulation cases the labeling could be questioned, and the labeling of suicides in the age group 11–15 in the Norwegian statistics, as represented in our material, seems to be closer to an overestimation rather than an underestimation, whereas none of the accidents gave any suspicion of incorrect labeling.

We know asphyxiation games have been played by individuals for generations [11]. These games are defined as self-strangulation or strangulation by another person for the purpose of achieving a brief euphoric state caused by cerebral hypoxia [45], without using drugs and not necessarily in a sexual context. In our dataset, we had two similar cases of strangulation, one labeled an accident and the other a suicide by SSB. Both were boys, alone in their room, sitting and leaned forward on their knees, with a noose around the neck, suffocated. Katz [46] preferred to call it “strangulation activity”, to indicate the potential danger of the game. Another question is how well does a child or an adolescent understand that strangulation can be lethal even with feet on solid ground.

Assessing grade of intent involves the rating of many aspects of the suicide, including the method, the understanding of the method’s lethality, location of the suicide, the risk of being interrupted during the act, the behavior prior to suicide, suicide note and/or other preparations, suicidal preoccupation and suicide threats. In addition to one’s mental health status, prior suicide attempts, personality traits, and the ability to cope with daily life and its challenges are important factors. In our sample, a suicide note was found in 41% of cases. The presence of suicide notes varies according to the literature, ranging from 4%-37% [20]. Copeland [47] found that 52% of 13-19-year-olds in his study had left notes. The numbers may be higher, as we cannot be certain if notes had been removed prior to the arrival of authorities or if letters were found later and thus not registered.

### Limitations

Psychological autopsies are always associated with recall bias. The respondent may tend to remember positive characteristics and forget the negative ones. Information may also be unreliable because the informant may be unaware of certain factors or may deliberately withhold information [48]. We achieved a low response rate of 45% in comparison with other autopsy studies that achieved closer to 70% [7,49,50]. This was a pervasive problem, making us cautious not to draw conclusions or generalize. In Table 2, due to many comparisons, all results ≥0.03 must be interpreted with great caution, to avoid Type I errors.

There is also the question as to who chose to participate in this type of research, and do these people differ from those who chose not to participate. The answers to these questions could possibly reveal a skewed distribution of the participant parents. We investigated two groups of deceased 15-year-olds and younger, dead by very different causes, which most likely colored the parents’ information. Concerning the personality traits, the use of The Junior Temperament and Character Inventory [51] would have made comparison to other studies possible. The interviews should ideally have been conducted closer to the actual time of the deaths, and interviews with teachers and friends could have provided additional and richer information. We obtained significantly fewer police reports in the accident group, which may have influenced our findings. However, the police reports we did have, gave very little information beyond the information provided by the parents.

### Ethical considerations

Asking parents to recall and talk about their deceased child, regardless of the cause of death, can be upsetting and can reactivate grief. We asked them to participate without being able to assure them that the data they provide will actually help prevent new deaths. However, we know from other similar studies that participation has commonly been perceived as a “positive” experience [52] and many of the interviewees seem to benefit from the interview [53].

## Conclusions

Suicide victims under the age of 16 resemble older adolescent suicide victims in most aspects, but with less apparent warning signs. We found that the extent of mislabeling between suicide and accident was small in our sample.

## Competing of interests

The authors declare that they have no competing interests.

## Authors’ contributions

B. Groholt and A. Freuchen contributed to the conceptualization of the study, were involved in the data analysis and contributed to the writing of the manuscript. A. Freuchen drafted the manuscript. E. Kjelsberg contributed to the writing of the manuscript. All authors read and approved the final manuscript.

## Acknowledgements

The Research Council of Norway and Sørlandet Sykehus HF funded the study and we received financial support from the Norwegian Directorate for Health and Social Affairs.
